# The causal relationship between circulating inflammatory proteins and heart failure: A two-sample Mendelian randomization study

**DOI:** 10.1097/MD.0000000000041115

**Published:** 2025-01-03

**Authors:** Fangxiang Wei, Haomiao Rui, Rutao Bian, Shunyu Liu

**Affiliations:** a The Second Clinical Medical College of Henan University of Chinese Medicine, Zhengzhou, China; b Henan Province Hospital of TCM (The Second Affiliated Hospital of Henan University of Chinese Medicine), Zhengzhou, China; c Zhengzhou Traditional Chinese Medicine Hospital, Zhengzhou, China.

**Keywords:** causality, circulating inflammatory proteins, FinnGen database, heart failure, Mendelian randomization

## Abstract

This study aims to explore the causal associations of 91 circulating inflammatory proteins with ischemic cardiomyopathy heart failure (ICM), dilated cardiomyopathy heart failure (DCM), and hypertrophic cardiomyopathy heart failure (HCM) to provide new ideas for the study of relevant heart failure mechanisms, adjunctive diagnosis and differentiation, and the clinical application of relevant drug targets. An analysis of the causal relationship between circulating inflammatory proteins and heart failure was conducted via inverse-variance weighted, weighted median estimator (WME), weighted mode (WM), and Mendelian randomization-Egger regression with Mendelian randomization. A Mendelian randomization analysis of 91 circulating inflammatory proteins revealed that natural killer cell receptor 2B4 levels, CXCL-6, fibroblast growth factor 5 levels, and interleukin-10 levels had positive causal relationships with ICM, whereas CX3CL-1, C-X-C motif chemokine 9 levels, interleukin-10 levels, leukemia inhibitory factor receptor levels, and signaling lymphocytic activation molecule levels had negative causal relationships; C-C motif chemokine 20 levels, C-X-C motif chemokine 5 levels, C-X-C motif chemokine 9 levels, fibroblast growth factor 5 levels, and oncostatin-M levels were positively correlated with DCM, whereas eukaryotic translation initiation factor 4E-binding protein 1 levels and Fms-related tyrosine kinase 3 ligand levels were negatively associated with DCM; and the CD40L receptor, Fms-related tyrosine kinase 3 ligand levels, hepatocyte growth factor levels, and sulfotransferase 1A1 levels were negatively associated with HCM. In this study, 9 of the 91 circulating inflammatory proteins were causally related to the ICM (4 positive, 5 negative), 7 were causally related to the DCM (5 positive, 2 negative), and 4 were causally related to the HCM (all negative). This study provides a theoretical foundation for the study of the relevant mechanisms of heart failure, clinical diagnosis, and treatment, as well as potential drug candidates closely related to heart failure.

## 1. Introduction

Heart failure (HF) is a complex group of clinical syndromes caused by abnormal changes in heart structure and function.^[1]^ Globally, >60 million people suffer from HF.^[2]^ The treatment of HF has been improved to the “five golden flowers” model,^[3]^ which can further reduce mortality and hospitalization risk and prolong patient survival. However, it has still not achieved satisfactory results.^[4,5]^ The most important etiological factors responsible for HF are myocardial injury and abnormalities, and ischemic cardiomyopathy (ICM), dilated cardiomyopathy (DCM), and hypertrophic cardiomyopathy (HCM) are the most common clinical conditions.^[6–8]^

Several studies have shown that the balance between inflammation and anti-inflammation is dysregulated in HF patients,^[9,10]^ and a wide range of circulating inflammatory markers are associated with their pathogenesis,^[11]^ auxiliary diagnosis,^[12]^ and drug target studies.^[13]^ Nevertheless, most existing studies have focused on certain inflammatory proteins and have yet to comprehensively assess the causal relationship between circulating inflammatory proteins and HF. Many confounding factors affect the accuracy of these studies.

In this study, we used Mendelian randomization (MR), which is an important research method in epidemiological research, with genetic variation as an instrumental variable (IV) to detect whether there is a causal relationship between exposure factors and outcomes, which can effectively avoid the influence of various confounding factors.^[14–16]^ In this study, the latest genome-wide data of 91 circulating inflammatory proteins were selected from the website of the Cardiovascular Epidemiology Unit of the University of Cambridge.^[17]^ To date, MR analysis has been used to evaluate the causal relationships of 91 circulating inflammatory proteins with osteoporosis, fractures, and degenerative diseases of the spine.^[18,19]^ However, the ICM, DCM, and HCM have not been investigated.

Our study aimed to explore the causal relationships between 91 circulating inflammatory proteins and 3 types of heart failure by applying MR analysis to several publicly available GWAS datasets and to provide new ideas for the study of HF-related mechanisms, the diagnosis and identification of auxiliary diseases, and the exploration of potential drug targets associated with these diseases.

## 2. Method

### 2.1. Study design

**Figure 1.**
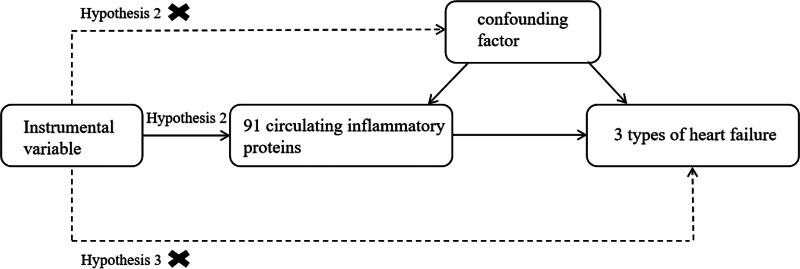
Model of the Mendelian randomization analysis.

In this study, several publicly released GWAS datasets were analyzed via 4 MR methods. A heterogeneity test, sensitivity analysis, and horizontal gene multiplicity test were used to verify the reliability of the test results. Methods for MR analysis must satisfy the 3 core assumptions of association, independence, and exclusivity at the same time: (1) the instrumental variable must be strongly correlated with the exposure factor; (2) the instrumental variable must not be correlated with any confounders associated with the exposure-outcome; and (3) the instrumental variable can influence the outcome only through the exposure factor (Fig. 1).

### 2.2. Data sources

The data for this study were derived from publicly available GWAS data for 91 circulating inflammatory proteins on the University of Cambridge Cardiovascular Epidemiology Unit website (n = 14, 824), as well as publicly accessible GWAS data for dilated cardiomyopathy (1, 444 cases, 353, 937 controls) and hypertrophic cardiomyopathy (507 cases, 489, 220 controls) on the GWAS Catalog website^[20]^ and ischemic heart disease GWAS data publicly accessible on the FinnGen website (49, 030 cases, 260, 124 controls).^[21]^ This study was based on publicly available data and did not require additional ethical approval (Table S1, Supplemental Digital Content, http://links.lww.com/MD/O257).

### 2.3. Selection of instrumental variables

Single-nucleotide polymorphisms (SNPs) significantly associated with 91 circulating inflammatory proteins were selected at the genome-wide level (*P* < 5 × 10^‐8^, r^2^ < 0.001, and genetic distance of 10,000 kb), and SNPs correlated with outcome (*P* < 5 × 10^‐5^) were filtered to ensure independence from each other. The PhenoScannerV2 database (https://ldlink.nih.gov/?tab=ldtrait)^[22]^ was searched to exclude SNPs correlated with potential confounders. The heterogeneity test was then utilized to exclude significantly heterogeneous SNPs, and finally, SNPs significantly associated with 91 circulating inflammatory proteins were determined as IVs. If the *F* value is <10, it indicates that there is a weak IV bias in the selected IVs, and then the relevant SNPs should be eliminated.^[23]^

### 2.4. Statistical analysis

In this study, we used inverse-variance weighted (IVW), weighted median estimator (WME), weighted mode (WM), and MR-Egger regression as the main analytical methods for MR analysis, with IVW as the primary analysis method and other methods as auxiliary analysis methods.^[24–26]^ The odds ratios (OR) values were used to assess the potential causal relationships between 91 circulating inflammatory proteins and HF. Furthermore, the validity and robustness of the IVW results were validated by testing for heterogeneity, sensitivity, and horizontal gene pleiotropy.

Heterogeneity tests were performed to determine the heterogeneity of SNPs through the Cochran *Q* test,^[27]^ which assesses the bias in the results due to measurement errors of SNPs for various reasons. The sensitivity analysis was performed via the leave-one-out method,^[28]^ whereby individual SNPs were sequentially deleted, and the combined effect value of the remaining SNPs was calculated, allowing us to evaluate the effect of each SNP on overall causality. The MR-PRESSO and MR-Egger regression intercept terms are commonly used as tests for horizontal genetic pleiotropy to ensure that IVs do not influence outcomes through any pathway other than exposure factors.^[29]^

The MR analyses involved in this study were performed via “TwoSampleMR”^[14–16]^ in R software. The final evaluation results are expressed as ORs and 95% confidence intervals (CIs), and the difference was considered statistically significant at *P* < .05.

## 3. Results

### 3.1. MR analysis of 91 circulating inflammatory proteins and ICM

**Figure 2.**
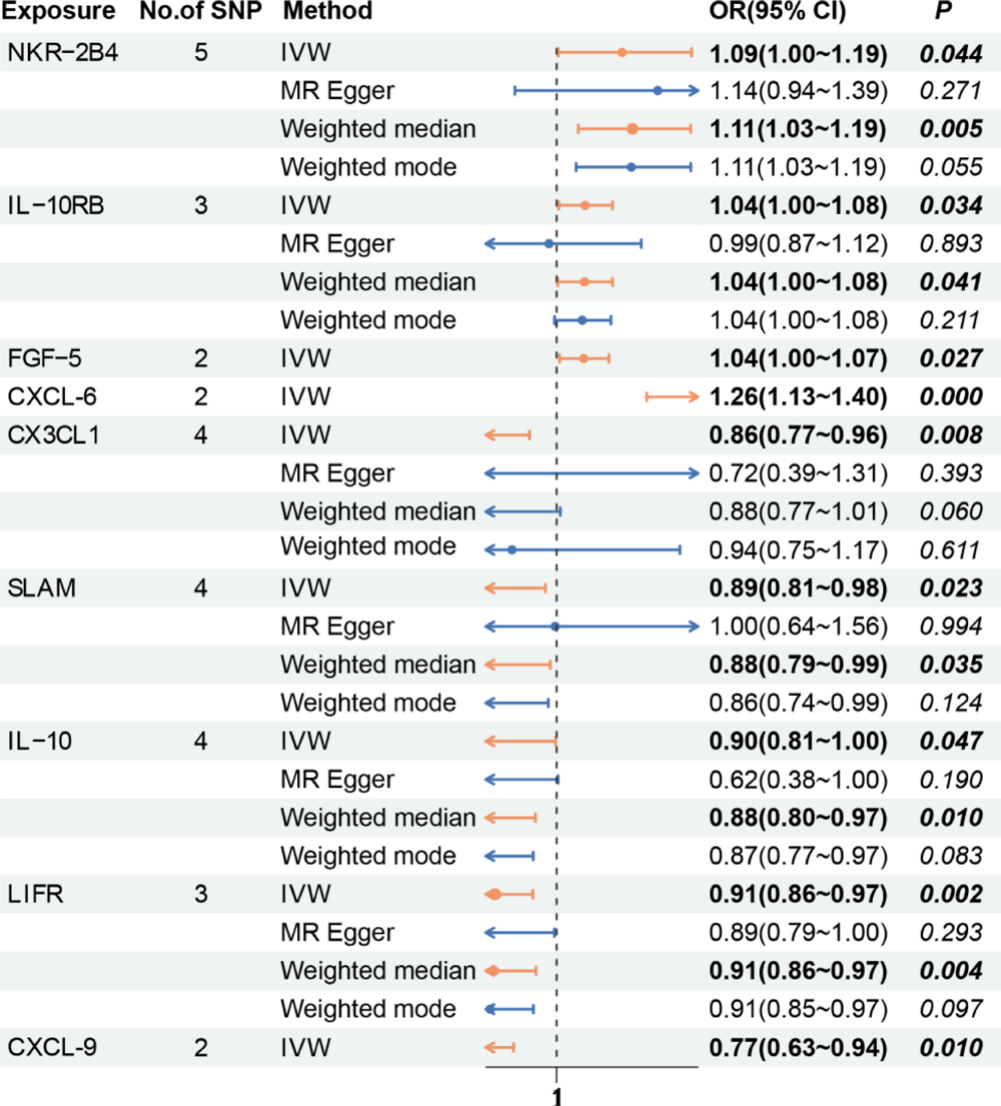
Effect values of the 4 MR methods corresponding to circulating inflammatory proteins with the significant causal relationships with the ICM. ICM = ischemic cardiomyopathy, MR = Mendelian randomization.

On the basis of the screening criteria for IVs in this study, 249 eligible SNPs were ultimately selected as IVs (*P* < 5 × 10^‐8^, r^2^ < 0.001), and the *F* statistics of all the SNPs included in the study were >10, demonstrating that there was no weak correlation with exposure. The analysis revealed a causal relationship between 9 circulating inflammatory proteins and the ICM (Table S2, Supplemental Digital Content, http://links.lww.com/MD/O257). The natural killer cell receptor 2B4 (NKR-2B4) (OR = 1.09, 95% CI: 1.00–1.19, *P* = .044), the chemokine CXCL-6 (OR = 1.26, 95% CI: 1.13–1.40, *P* = .000), the fibroblast growth factor 5 (FGF-5) (OR = 1.04, 95% CI: 1.00–1.07, *P* = .027), and the interleukin-10 receptor subunit beta (IL-10RB) (OR = 1.04, 95% CI: 1.00–1.08, *P* = .034) were positively causally related to the ICM, whereas the chemokine fractalkine (Fkn, CX3CL1) (OR = 0.86, 95% CI: 0.77–0.96, *P* = .008), the chemokine C-X-C motif chemokine 9 levels (CXCL-9) (OR = 0.77, 95% CI: 0.63–0.94, *P* = .010), and the interleukin-10 (IL-10) (OR = 0.90, 95% CI: 0. The number of SNPs was 5 SNP_NKR-2B4_, 4 SNP_CX3CL-1_, 2 SNP_CXCL-6_, 2 SNP_CXCL-9_, 2 SNP_FGF-5_, 4 SNP_IL-10_, 3 SNP_IL-10RB_, 3 SNP_LIFR_ and 4 SNP_SLAM_ (Fig. 2). The Cochran *Q* test results *revealed P values* > .05, and no significant heterogeneity was detected. The leave-one-out test results *revealed P values* < .05, suggesting that when each SNP was removed in turn, the remaining SNPs were similar to those in the overall analysis. MR-PRESSO did not detect any abnormalities, and the *P values* were >.05, suggesting no statistical significance; thus, there was no need to consider the effect of horizontal gene diversity on the results except for a few exposures with no relevant data (Table S3, Supplemental Digital Content, http://links.lww.com/MD/O257).

### 3.2. MR analysis of 91 circulating inflammatory proteins and DCM

**Figure 3.**
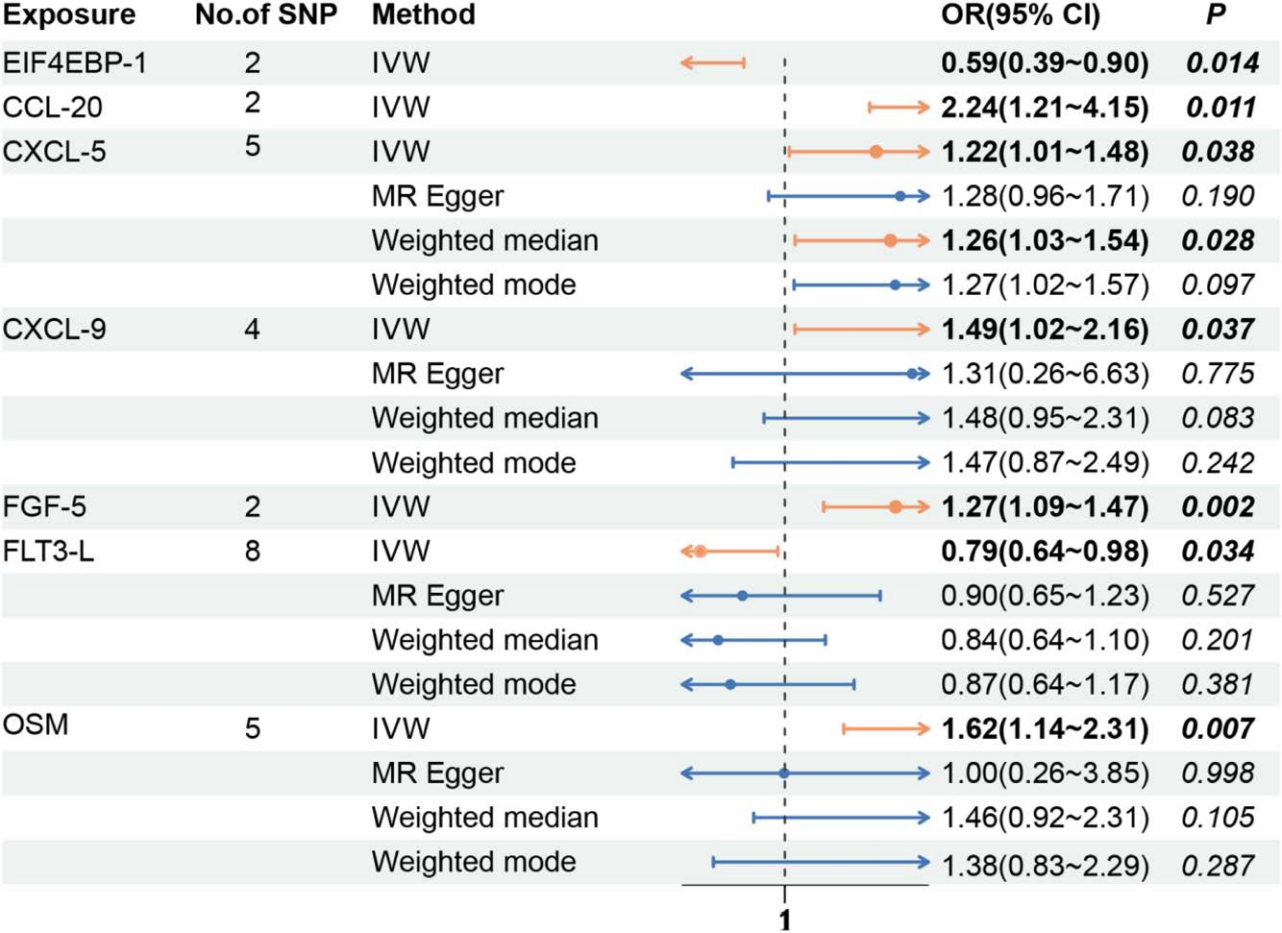
Effect values of 4 MR methods corresponding to circulating inflammatory proteins with a significant causal relationship with DCM. DCM = dilated cardiomyopathy, MR = Mendelian randomization.

On the basis of the screening criteria for IVs in this study, 278 eligible SNPs were ultimately selected as IVs (*P* < 5 × 10^‐8^, r^2^ < 0.001), and the *F* statistics of all the SNPs included in the study were >10, demonstrating that there was no weak correlation with exposure. The results of the analysis revealed a causal relationship between 7 circulating inflammatory proteins and DCM (Table S4, Supplemental Digital Content, http://links.lww.com/MD/O257). The chemokines C-C motif chemokine 20 levels (CCL-20) (OR = 2.24, 95% CI: 1.21–4.15, *P* = .011), C-X-C motif chemokine 5 levels (CXCL-5) (OR = 1.22, 95% CI: 1.01–1.48, *P* = .038), CXCL-9 (OR = 1.49, 95% CI: 1.02–2.16, *P* = .037), FGF-5 (OR = 1.27, 95% CI: 1.09–1.47, *P* = .002), oncostatin-M (OSM) (OR = 1.62, 95% CI: 1.14–2.31, *P* = .007) were positively causally related to DCM, whereas the eukaryotic translation initiation factor 4E-binding protein 1 (EIF4EBP-1) (OR = 0.59, 95% CI: 0.39–0.90, *P* = .014) and Fms-related tyrosine kinase 3 ligand (FLT3-L) (OR = 0.79, 95% CI: 0.64–0.98, *P* = .034) were negatively causally related to DCM. The number of SNPs was 2 SNP_EIF4EBP-1_, 2 SNP_CCL-20_, 5 SNP_CXCL-5_, 4 SNP_CXCL-9_, 2 SNP_FGF-5_, 8 SNP_FLT3-L_, and 5 SNP_OSM_ (Fig. 3). The Cochran *Q* test results *revealed P values* > .05, and no significant heterogeneity was detected. The leave-one-out test results *revealed P values* < .05, suggesting that when each SNP was removed in turn, the remaining SNPs were similar to those in the overall analysis. MR-PRESSO did not detect any abnormalities, and the *P values* were >.05, suggesting no statistical significance; thus, there was no need to consider the effect of horizontal gene diversity on the results except for a few exposures with no relevant data (Table S5, Supplemental Digital Content, http://links.lww.com/MD/O257).

### 3.3. MR analysis of 91 circulating inflammatory proteins and HCMs

**Figure 4.**
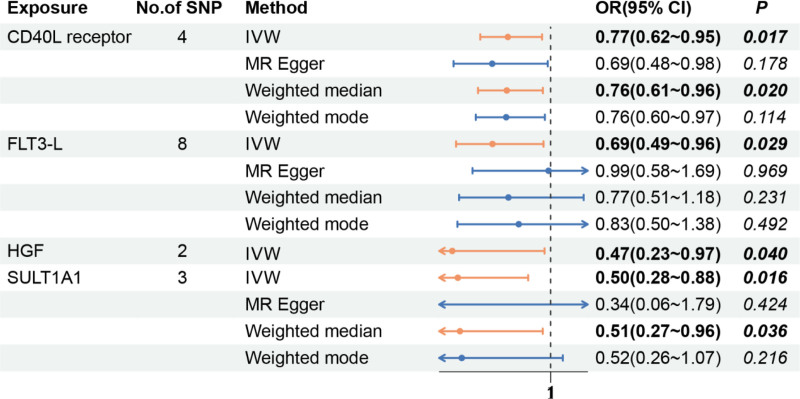
Effect values of the 4 MR methods corresponding to circulating inflammatory proteins with a significant causal relationship with HCM. HCM = hypertrophic cardiomyopathy, MR = Mendelian randomization.

On the basis of the screening criteria for IVs in this study, 280 eligible SNPs were ultimately selected as IVs (*P* < 5 × 10^‐8^, r^2^ < 0.001), and the *F* statistics of all the SNPs included in the study were >10, demonstrating that there was no weak correlation with exposure. A negative causal relationship was identified between HCMs and 4 circulating inflammatory proteins: the CD40L receptor levels (CD40L receptor) (OR = 0.77, 95% CI: 0.62–0.95, *P* = .017), FLT3-L (OR = 0.69, 95% CI: 0.49–0.96, *P* = .029), hepatocyte growth factor (HGF) (OR = 0.47, 95% CI: 0.23–0.97, *P* = .040), and human sulfotransferase 1A1 (SULT1A1) (OR = 0.50, 95% CI: 0.28–0.88, *P* = .016), and no positive causal relationship was found (Table S6, Supplemental Digital Content, http://links.lww.com/MD/O257). The number of SNPs was 4 SNP_CD40L receptors_, 8 SNP_FLT3-L_, 2 SNP_HGF_, and 3 SNP_SULT1A1_ (Fig. 4). The Cochran *Q* test results *revealed P values* > .05, and no significant heterogeneity was detected. The leave-one-out test results *revealed P values* < .05, suggesting that when each SNP was removed in turn, the remaining SNPs were similar to those in the overall analysis. MR-PRESSO did not detect any abnormalities, and the *P values* were >.05, suggesting no statistical significance; thus, there was no need to consider the effect of horizontal gene diversity on the results except for a few exposures with no relevant data (Table S7, Supplemental Digital Content, http://links.lww.com/MD/O257).

## 4. Discussion

On the basis of the results of this study, 9 of the 91 circulating inflammatory proteins were causally associated with ICM, of which 4 were positively correlated, and 5 were negatively correlated; 7 were causally associated with DCM, of which 5 had positive causality, and 2 were negatively related; and 4 were negatively related with HCM.

Recent studies have associated several chemokines with cardiovascular disease.^[30,31]^ CXCL-9 is closely associated with the development of cardiomyopathy,^[32]^ which may increase the risk of acute cardiovascular events during hospitalization,^[33]^ elucidating, to some extent, the existence of a positive causal relationship between DCM and CXCL-9. According to the present study, there is a negative causal relationship between it and ICM. A further investigation of the specific reasons is necessary. The interleukin family is strongly associated with cardiovascular disease,^[34]^ and IL-10 can improve cardiac function by suppressing inflammation.^[35]^ Our findings revealed a negative causal relationship between IL-10 and ICM, which is consistent with the findings of previous studies. Previous MR studies have demonstrated that FGF-5 is causally associated with cardiovascular disease risk,^[36]^ which is consistent with the results of the present study. Notably, the present study revealed a positive causal relationship between FGF-5 and both ICM and DCM, which may be a common risk factor for these 2 types of heart failure. FLT3-L has also been found to have a negative causal relationship with both DCM and HCM, possibly because it provides cardiomyocyte protection and improves cardiac function.^[37]^ A number of studies have demonstrated that leukemia inhibitory factor receptor levels (LIFR) is primarily found in the interstitium of the myocardium of hypertrophic hearts and that it is closely associated with cardiac hypertrophy.^[38]^ This study, however, did not find a causal relationship between LIFR and DCM or HCM; rather, it found a negative causal relationship with ICM, which deserves further investigation.

Previous studies have suggested that OSM is a major mediator of myocardial remodeling and that sustained high levels of OSM promote the development of DCM,^[39]^ which was confirmed by the finding of a positive causal relationship between OSM and DCM in the present study. In an animal study, signaling lymphocytic activation molecule levels (SLAM) was found to be highly expressed in the heart.^[40]^ However, few studies have investigated the link between SLAM and CVD, and the present study revealed a negative causal relationship between SLAM and ICM, demonstrating that SLAM may limit the development of ICM to a certain extent, which provides ideas for further research. In addition, a recent study reported that higher concentrations of HGF resulted in increased ventricular volume and deterioration of cardiac function,^[41]^ which contradicted the results of the present study, which revealed that there was a negative causal relationship between HGF and HCM but not between ICM and DCM. The exact reasons for this need to be explored in broader detail.

In addition, this study demonstrated for the first time that NKR-2B4, CXCL-6, and IL-10RB have positive causal relationships with ICM and that CX3CL-1 has a negative causal relationship with ICM. CCL-20 and CXCL-5 have positive causal relationships with DCM, whereas EIF4EBP-1 and DCM have negative causal relationships. The CD40L receptor and SULT1A1 have negative causal relationships with HCM. Nevertheless, few studies have been conducted on this topic, and more research is needed on the specific mechanisms involved.

This study has the following advantages: ① The MR analysis method was used to avoid confounding factors and reverse causality from interfering with the results of the study by using genetic variation as an instrumental variable. As genetic variation is stable and alleles are randomly assigned, the reliability of the study’s results may be improved. ② Compared with previous studies on circulating inflammatory proteins, this study utilized the most recent publicly available data on 91 circulating inflammatory proteins, which are more comprehensive than those of previous studies. However, there are several limitations in this study: ① the GWAS data adopted in this study were mainly from European populations, and further validation in Asian populations is needed in the future; ② this study categorized HF according to the etiology of HF and chose the GWAS data of ICM, DCM, and HCM as the study subjects, which are the common clinical etiologies of HF.^[6–8]^ In the future, it is necessary to obtain more detailed and accurate GWAS data on various types of HFs. These findings will enable a more comprehensive exploration of the causal relationships between circulating inflammatory proteins and different types of HF.

Using multiple GWAS data, this study examined the causal relationships between 91 circulating inflammatory proteins and various types of heart failure. These findings lay a theoretical foundation for the study of HF-related mechanisms, clinical diagnosis, and treatment and are expected to reveal potential drug targets closely related to HF.

## Acknowledgments

We thank the participants in all the GWASs used in this study and the investigators who made these GWAS data publicly available. We want to thank the participants and researchers of the FinnGen study.

## Author contributions

**Data curation:** Rutao Bian.

**Methodology:** Fangxiang Wei, Rutao Bian.

**Supervision:** Haomiao Rui.

**Writing – original draft:** Fangxiang Wei.

**Writing – review & editing:** Haomiao Rui, Rutao Bian, Shunyu Liu.

## Supplementary Material


